# Socioeconomic determinants of geographic disparities in campylobacteriosis risk: a comparison of global and local modeling approaches

**DOI:** 10.1186/1476-072X-11-45

**Published:** 2012-10-13

**Authors:** Jennifer Weisent, Barton Rohrbach, John R Dunn, Agricola Odoi

**Affiliations:** 1 Department of Biological and Diagnostic Sciences, College of Veterinary Medicine, The University of Tennessee, 2407 River Drive, Knoxville, TN, 37996, USA; 2 Tennessee Department of Health, Communicable and Environmental Disease Service, 1st Floor, Cordell Hull Bldg. 425 5th Ave. North, Nashville, TN, 37243, USA

**Keywords:** Campylobacter, Socioeconomic determinants, Geographically weighted regression, Spatial modeling

## Abstract

**Background:**

Socioeconomic factors play a complex role in determining the risk of campylobacteriosis. Understanding the spatial interplay between these factors and disease risk can guide disease control programs. Historically, Poisson and negative binomial models have been used to investigate determinants of geographic disparities in risk. Spatial regression models, which allow modeling of spatial effects, have been used to improve these modeling efforts. Geographically weighted regression (GWR) takes this a step further by estimating local regression coefficients, thereby allowing estimations of associations that vary in space. These recent approaches increase our understanding of how geography influences the associations between determinants and disease. Therefore the objectives of this study were to: (i) identify socioeconomic determinants of the geographic disparities of campylobacteriosis risk (ii) investigate if regression coefficients for the associations between socioeconomic factors and campylobacteriosis risk demonstrate spatial variability and (iii) compare the performance of four modeling approaches: negative binomial, spatial lag, global and local Poisson GWR.

**Methods:**

Negative binomial, spatial lag, global and local Poisson GWR modeling techniques were used to investigate associations between socioeconomic factors and geographic disparities in campylobacteriosis risk. The best fitting models were identified and compared.

**Results:**

Two competing four variable models (Models 1 & 2) were identified. Significant variables included race, unemployment rate, education attainment, urbanicity, and divorce rate. Local Poisson GWR had the best fit and showed evidence of spatially varying regression coefficients.

**Conclusions:**

The international significance of this work is that it highlights the inadequacy of global regression strategies that estimate one parameter per independent variable, and therefore mask the true relationships between dependent and independent variables. Since local GWR estimate a regression coefficient for each location, it reveals the geographic differences in the associations. This implies that a factor may be an important determinant in some locations and not others. Incorporating this into health planning ensures that a needs-based, rather than a “one-size-fits-all”, approach is used. Thus, adding local GWR to the epidemiologists’ toolbox would allow them to assess how the impacts of different determinants vary by geography. This knowledge is critical for resource allocation in disease control programs.

## Background

*Campylobacter* organisms are leading causes of human gastroenteritis in developed nations, affecting an estimated 13 million people in the United States annually [1,2]. Campylobacteriosis risk (defined as the probability that an individual will develop campylobacteriosis within a given time period [3]) is known to vary by geographic regions, climate patterns, human behaviors, and food and water sources [4-6]. The Foodborne Diseases Active Surveillance Network (FoodNet) has reported substantial geographic variations in the risk of *Campylobacter* infections across the U.S. Currently, the reasons for these variations are unknown. Although the overall risk of disease in the U.S. population is 13 cases per 100,000, some areas in Tennessee have risks as high as 200 cases per 100,000 [7]. Therefore, there is interest in identifying the determinants of the geographic disparities seen in disease risk so as to guide disease control efforts.

Understanding health disparities among racial, ethnic and across socioeconomic subgroups is a priority of public health personnel and policy makers [8,9]. In developed countries, the relationships between socioeconomic status (SES) and campylobacteriosis risk are complex and have been shown to vary geographically. For example, disease risks were found to be significantly lower in rural compared to urban areas in several studies [10-12]. This is possibly due to continued exposure in rural areas, resulting in sustained immunity and hence lower disease incidence [13]. However, in Manitoba, Canada the risk for young children was seven times higher in rural regions than in the City of Winnipeg [14]. In a New Zealand study, urban adults and rural children had higher rates of disease, signifying an age based disparity between these areas [10]. The geographic differences in associations between SES factors and campylobacteriosis risk are a worldwide phenomenon, hence regression models need to account for the fact that regression coefficients, used to assess associations between risk factors and disease, might vary in space. Local modeling approaches enable investigators to more accurately estimate the true relationships between determinants and disease risk since they estimate regression coefficients for each location in the study area [15-17].

Geographically weighted regression (GWR) modeling techniques compute local regression coefficients thereby allowing the estimates of the associations between outcome and explanatory variables to vary spatially [18,19]. This flexible modeling strategy is necessary to improve our understanding of the determinants of geographic disparities of campylobacteriosis risk internationally. Recent interest in exploring geographic variation in the associations between socioeconomic factors and health outcomes has spurred studies in the US, UK and Taiwan. For example, local variations were detected in the occurrence of diseases such as obesity and breast cancer when modeled against socioeconomic factors [20-22]. In these and other studies, global modeling approaches, that estimate one regression coefficient for each variable in the model, hide local variations in associations [16,17]. Since a local GWR model estimates a regression coefficient of an explanatory variable for each location in the study area [18], it allows health professionals to better assess how the effect of the explanatory variable changes by geographic location. Armed with this knowledge, health planners can better identify the most important disease determinants for different regions and therefore better plan health programs, provision of services and resource allocation to meet the unique needs of different communities. Thus, this helps health professionals avoid using a one-size-fits-all approach but instead use empirical evidence provided by the local GWR models, to practice needs-based population health planning, enabling them to provide services based on the unique health needs of the different populations they serve. Thus the objectives of this study were to: (i) identify socioeconomic determinants of the geographic disparities of campylobacteriosis risk at the census tract level (ii) investigate whether regression coefficients for the associations between socioeconomic factors and campylobacteriosis risk demonstrate spatial variability and (iii) compare the performance of negative binomial, spatial lag and global and local Poisson GWR models.

## Methods

### Study area and data sources

The study was conducted in the state of Tennessee which consists of 1,261 census tracts, with an estimated total population of 5.7 million in 2000 [23]. Census tracts are ‘statistical geographic entities’ which typically contain between 2,500 and 8,000 people and are relatively homogeneous with respect to population characteristics and socioeconomic conditions [23].

Cartographic boundary files, population denominator data, including the U.S. standard population used for risk standardization, and socioeconomic variables were obtained, at the census tract level, from the year 2000 U.S. Census [23]. Campylobacteriosis data were collected through the FoodNet active surveillance system [24] and obtained from the Tennessee Department of Health. These data covered the period from September 1, 1991 to December 31, 2008. Cases of campylobacteriosis were defined as culture-confirmed *Campylobacter* infections from clinical specimens, the majority of which were stool samples. Culture results were reported at the genus level only. Names of patients were deleted from the database before it was released to investigators. The study was approved by Tennessee Department of Health and The University of Tennessee Institution Review Boards.

### Geocoding and data aggregation

The dataset consisted of a total of 4,723 confirmed campylobacteriosis cases reported during the study period. Initial data exploration, preparation and formatting for geocoding, was performed in SAS version 9.2 [25]. Cases that could not be geocoded for various reasons (see Table 1) or that were due to infections acquired outside of the study area (travel-related) were excluded from the dataset.

**Table 1:** Cases deleted from initial dataset and reasons for deletion.

**Number of cases (total=967)**	**% Total**	**Reason for removal**
130	2.75	Infection acquired outside study area (travel-related)
40	0.85	No address provided
24	0.51	Duplicate data/data entry error
4	0.05	Missing sex
135	2.80	Missing age
520	11.00	Missing street or address information
244	5.20	Incorrect address or typographical error

Note: One hundred twenty nine cases contained more than one of the above reasons for removal. (Total number of cases described above=1096, Total number eliminated=967).

An iterative geocoding process was performed to accurately match location data to the finest possible geographic scale using both Googlemaps [26] and Yahoo Maps Geocoder through BatchGeo [27]. Dot maps of the final dataset were generated in ArcGIS [28] and included 3,756 cases: 2,638 (70%) rooftop accuracy and 1,118 (30%) street level accuracy. The geocoding was classified as ‘rooftop’ accuracy when there was a 100% match to the address. The ‘street level’, also known as ‘range interpolated’ accuracy, referred to instances when interpolation was used to identify address location along a street. This was done in cases where the exact street address number was unavailable in the geocoding database. The campylobacteriosis data were then aggregated to the census tract level for subsequent analyses.

### Computation of campylobacteriosis risk, smoothing and mapping

The measure of disease frequency used as the dependent variable in this study was campylobacteriosis risk. Census tract campylobacteriosis risk, for the entire 17 year study period, was computed as the number of campylobacteriosis cases reported in a census tract during the study period divided by the US 2000 census population of the census tract. However, due to the potential confounding effect of age and sex, campylobacteriosis risk was age and sex standardized using STATA version 9.0 [29]. This ensured that differences in geographic distribution of campylobacteriosis risks observed were not due to geographical differences in the distribution of age and/or sex of the population. The computed age and sex standardized campylobacteriosis risks (for the entire 17 year study period) were then presented as number of campylobacteriosis cases per 100,000 people/population. Socioeconomic variables from the 2000 U.S. Census, at the census tract level, were then merged to the age and sex standardized risk estimates [23] and spatial empirical Bayes smoothing was performed in GeoDa version 095i [30]. Jenk’s optimization classification method was used to determine critical intervals for spatial display of maps in ArcView [28]. The unsmoothed campylobacteriosis risk estimates and the socioeconomic variables of interest were then assessed for spatial autocorrelation at the census tract level using Global Moran’s I in GeoDa [30].

### Regression analysis

#### Univariate regression analysis

Race and ethnicity variables investigated for potential association with geographic distribution of campylobacteriosis risk were the proportion of the population that were black, white, Asian, Chinese, Hispanic/Latino, and Native American/Alaskan. Employment related variables included the proportion of the population that were unemployed, and those whose occupations were in the farming, fishing, forestry or service industries. Variables related to marital status included the proportion of the population that was divorced, never married, separated, or widowed. Educational attainment factors included the proportion of the population with no high school diploma, and those with a bachelor’s or graduate degree. Other socioeconomic variables investigated were: the proportion of the population living in rural verses urban areas, those in the armed services, those who were disabled, living in poverty or those on public assistance.

Pairwise Spearman rank correlation coefficients were computed to identify highly correlated explanatory/independent variables. Using a cut-off Spearman rank correlation coefficient of 0.6, only one of a pair of highly correlated variables (i.e. with r≥0.6) was retained for further investigation. Univariate (or simple) ordinary Poisson models were fit to the data using the generalized linear model procedure, PROC GENMOD, in SAS [25]. The dependent variable specified in the model was the number of campylobacteriosis cases reported in each census tract and the offset was specified as the census tract population derived from the 2000 US census. Although we recognize that the 2000 US census population is not ideal since the population must have changed during the study period, it was the only population data available for the study time period. Suffice it to say that the year 2000 is the mid-point of the study period and therefore the census tract population at this time is assumed to approximately represent the average population over the study period. An assessment of overdispersion revealed significant overdispersion of the univariate (simple) ordinary Poisson models implying that the ordinary Poisson models were inappropriate for the data. Therefore, negative binomial models were used for all subsequent multivariable modeling and final comparisons with other modeling approaches investigated in the study.

### Multivariable regression analyses

#### Negative binomial models

As for the ordinary Poisson models, the dependent variables specified in the multivariable negative binomial models were the number of campylobacteriosis cases reported in each census tract and the offset variable was again the census tract population derived from the 2000 US census. The regression equation for the negative binomial model is:

$$1n\left(\lambda \right)=\beta_{0}+\beta_{1}X_{1}+\beta_{2}X_{2}+\ldots +\beta_{n}X_{n}$$

Where λ is E(Y)/n, Y is the dependent variable, the βs are parameter estimates (regression coefficients) and the Xs are the socio-economic variables under investigation.

Initial multivariable negative binomial model building was performed using the forward stepwise selection procedure in SAS, using likelihood ratio tests to assess significance of variables in the model. Throughout the selection procedure, previously removed variables remained eligible for re-entry into the model, provided they were statistically significant (p<0.05) and improved Akaike’s Information Criterion (AIC) by three or more points. Only one of a pair of highly correlated variables, such as black and white race, and rural or urban locale were entered into the model in order to avoid issues of collinearity.

McHenry’s All Possible selection method in NCSS [31] in conjunction with the SAS model comparison macro, %genmodsummary [25], were used to identify the two most parsimonious multivariable models. Two-way interaction terms on the variables included in the main effects models were assessed for significance and model improvement. Residual diagnostics were performed by investigating for outliers (using standardized Pearson’s residuals) and influential points (using Cook’s Distance). Both the raw and standardized Pearson’s residuals were also assessed for spatial autocorrelation using Global Moran’s I [30].

#### Spatial Lag models

To account for spatial autocorrelation in the residuals (identified in the negative binomial models), a spatial lag model was fit to the data in GeoDa [30], specifying the log transformed campylobacterisis risk as the dependent variable. The equation for the spatial lag model is:

$$Y=\rho WY+\beta_{0}+\beta_{1}X_{1}+\beta_{2}X_{2}+\ldots +\beta_{n}X_{n}$$

Where Y is the dependent variable (log transformed campylobacteriosis risk), ρ is the spatial autoregressive coefficient of the spatial lag model, W is the spatial weight, WY is the spatial lag for the dependent variable, the βs are parameter estimates (regression coefficients) and the Xs are the socio-economic variables under investigation. A significant ρ of this model means presence of significant spatial autocorrelation of the dependent variable implying that a non-spatial model (such as the negative binomial model) is inappropriate for the data. The autoregressive coefficient is also an estimate of the degree of spatial autocorrelation present in the data. Using the queen definition of neighborhood contiguity, a correlogram was constructed to identify the most optimal spatial weight. The queen spatial weights assessed were from 1^st^ to 5^th^ order, with each weight construction including lower orders. For instance, 2^nd^ order queen weights included both 1^st^ and 2^nd^ order neighbors and 3^rd^ order weights included 1^st^, 2^nd^ and 3^rd^ order neighbors in the construction of the spatial weight. The 3^rd^ order Queen weight resulted in the best model fit and eliminated residual spatial autocorrelation. Since the campylobacteriosis risk and the proportion of the population that were black required log transformation, a factor of 1.1 was added to all records of these variables to deal with zero values.

Model residuals were assessed for normality (using Jarque-Bera and White tests), homoskedasticity (using Breusch-Pagan test) and residual spatial autocorrelation (using Moran’s I). Additionally, the statistical significance of the spatial autoregressive coefficient was assessed using the likelihood ratio test.

#### Global and local Poisson geographically weighted (GWR) models

Global models estimate one coefficient per explanatory variable, averaged over all locations, whereas local Geographically Weighted Regression (GWR) models estimate as many coefficients as the number of locations (in this case, census tracts) in the dataset. The equation for the local GWR model is:

$$Y_{i}\left(\mu \right)=\beta_{0i}\left(\mu \right)+\beta_{1i}\left(\mu \right)X_{1i}+\beta_{2i}\left(\mu \right)X_{2i}+\ldots +\beta_{\mathrm{ni}}\left(\mu \right)X_{\mathrm{ni}}$$

Where the β_ni_(u) are regression coefficients for the relationship between an explanatory variable and the dependent variable around a location u and is therefore unique to that location while the Xs are the different explanatory/independent variabes included in the model.

The local GWR model allows the investigator to compute a different regression coefficient for each location when assessing the relationship between the dependent and independent variable. Thus, assuming a causal relationship, it enables the investigator to assess how the impact of a specific risk factor on the outcome changes by location. The Poisson distribution within the GWR framework is currently the most appropriate available strategy for analyzing areal disease counts, especially when low numbers are involved [18,19]. As in the negative binomial models, the dependent variables for both the global and local Poisson GWR models were the number of cases of campylobacteriosis in a census tract and the offset variable was population of the census tract. Both models were fit in a specialized spatial statistical software called GWR [32].

For the local Poisson GWR, the adaptive kernel method was chosen to account for differences in the density of census tracts across the study area. Accommodating irregularly shaped census tracts is particularly important, as shapes, sizes and density varies widely between metropolitan and rural regions. The kernel varies the size of the analysis window so as to incorporate the same number of census tracts in each local estimate. In each local regression analysis a zero weight value is applied to all other census tracts not included in the analysis window. A manual iterative approach identified 300 nearest neighbors (census tracts) as the optimal model bandwidth based on AIC [19,32].

To demonstrate spatial variability in the association between campylobacteriosis risk and the explanatory variables, the estimated regression coefficients from the local GWR were displayed as choropleth maps using Jenk’s optimization classification scheme. Assessment of goodness-of-fit of the negative binomial, spatial lag, global and local Poisson GWR models was done using AIC.

## Results

### Spatial distribution of campylobacteriosis risk and socioeconomic factors

**Figure 1:**
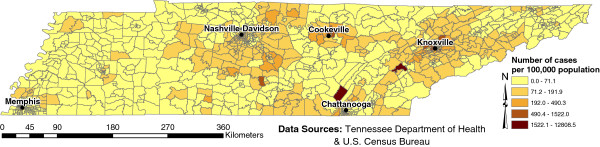
**Age & sex standardized spatial empirical Bayes smoothed risk of campylobacteriosis in Tennessee at the census tract level.** Figure adopted from Geospatial Health;6(1):65–76.

Age and sex adjusted campylobacteriosis risk estimates varied widely, ranging from 0 (n=241, median=53.8) to 13,122 per 100,000 population. The spatial empirical Bayes smoothed map of campylobacteriosis risk showed evidence of geographic disparities in risk across the study area (Figure 1, adopted from our previous study published in Geospatial Health;6(1):65–76, used with permission). Areas of high campylobacteriosis risk centered around the cities of Knoxville, Cookeville, south of metropolitan Nashville-Davidson and north of Chattanooga [7].

**Figure 2:**
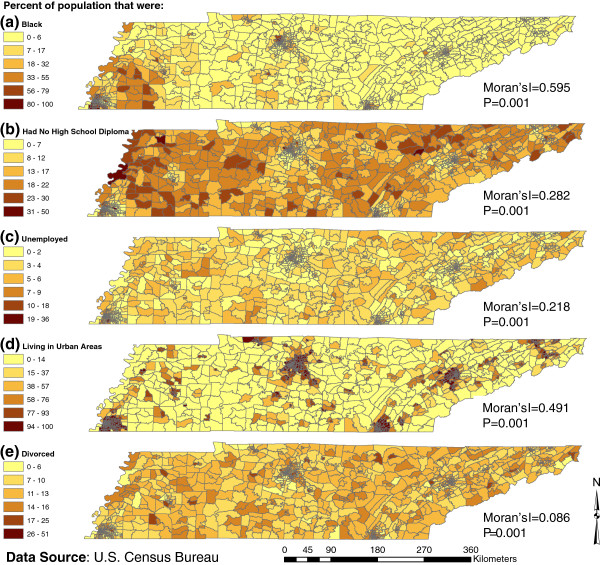
Geographic distribution of selected socioeconomic variables investigated for potential association with campylobacteriosis risk in Tennessee.

Summary statistics of the selected socioeconomic factors showed that the study area is predominantly inhabited by whites followed by black, Hispanic/Latino and Asian residents (Table 2). In one census tract up to 100% of the population was reported to be living in poverty and approximately 50% had no high school diploma. Spatial distributions of the socioeconomic factors under investigation showed evidence of spatial clustering based on the statistically significant (p=0.001) Moran’s I statistics (Figure 2). Census tracts in and around Memphis had high proportions of black population (Figure 2a), whereas rural census tracts tended to have higher proportions of the population with no high school diploma (Figure 2b).

**Table 2:** Summary statistics of the socioeconomic factors investigated for potential associations with campylobacteriosis risk

**Category**	**Variable (% of census tract population)**	**Mean**	**Std Dev**	**Median**	**Min**	**Max**
Race & Nationality	Black or African American	19.2	28.4	5.6	0	99.7
	White	77.2	29.0	91.1	0	100.0
	American Indian or Alaskan household	0.3	0.4	0.2	0	11.1
	Asian	1.0	1.7	0.4	0	25.6
	Hispanic/Latino	2.1	2.9	1.2	0	34.2
	Native American/Alaskan	0.2	0.2	0.2	0	1.1
Employment	Unemployed	3.8	2.9	3.2	0	36.3
	Service occupation	14.9	6.7	13.6	0	82.6
	Agriculture: forestry, fish/hunt/mine	1.6	2.3	0.7	0	20.4
	Farming Industry	0.6	1.1	0.3	0	14.5
	Disability (age 21–64)	23.1	8.6	22.9	0	60.4
	Armed forces	0.3	2.7	0.0	0	70.1
Education	No high school diploma	15.7	7.7	15.8	0	50.2
	Bachelor degree	11.9	9.3	8.7	0	52.1
	Graduate or Professional Degree	6.4	6.2	4.3	0	39.6
Marital status	Never married	24.4	11.1	19.9	0	88.9
	Separated	2.3	2.2	1.7	0	21.7
	Divorced	11.6	3.9	11.3	0	51.1
	Widow	7.4	3.3	7.0	0	28.5
Poverty, Public assistance & Urbanicity	Poverty level	12.5	11.3	9.7	0	100.0
	Receive public assistance	4.0	4.4	2.8	0	57.1
	Urban	62.7	42.8	89.1	0	100.0
	Rural	37.1	42.7	10.0	0	100.0

Data source: U.S. Census 2000.

Significant positive correlations were found between the proportion of the population that were black and those that were never married (r=0.73; p<0.001) or that were separated (r=0.61; p<0.001) (Table 3). As expected, the proportion of the census tract population employed in the agricultural industry was highly correlated with the proportion of the population living in rural regions (r=0.78; p<0.001). Census tracts with a high proportion of those with a bachelor’s degree tended to have lower proportions of those who were living in poverty (r=−0.61; p<0.001) and those with no high school diploma (r=−0.79; p<0.001).

**Table 3:** Spearman Rank Correlation Coefficients of variables investigated for potential association with campylobacteriosis in Tennessee

	**Black**	**White**	**Unemp**^**1**^	**Service Industry**	**Ag Employ**^**2**^	**Dis-ability**	**No HS Diploma**^**3**^	**Bach Degree**^**4**^	**Grad Degree**^**5**^	**Never Married**	**Sep**^**6**^	**Divorced**	**Poverty**	**Public Assist**^**7**^	**Urban**	**Rural**
Black	1															
White	−0.97	1														
	(<.001)															
Unemployed	0.37	−0.35	1													
	(<.001)	(<.001)														
Service Industry	0.40(<.001)	−0.39(<.001)	0.57(<.001)	1												
Ag Employ^2^	−0.49	0.52	−0.07	−0.17	1											
	(<.001)	(<.001)	(<.001)	(<.001)												
Disability	0.15	−0.11	0.51	0.55	0.19	1										
	(<.001)	(<.001)	(<.001)	(<.001)	(<.001)											
No High School Diploma	0.20(<.001)	−0.15(<.001)	0.50(<.001)	0.51(<.001)	0.24(<.001)	0.76(<.001)	1									
Bachelor’s Degree	0.05(0.08)	−0.06(0.02)	−0.39(<.001)	−0.35(<.001)	−0.41(<.001)	−0.72(<.001)	−0.79(<.001)	1								
Graduate Degree	−0.01(0.86)	−0.01(0.61)	−0.36(<.001)	−0.32(<.001)	−0.34(<.001)	−0.62(<.001)	−0.73(<.001)	0.84(<.001)	1							
Never Married	0.73(<.001)	−0.74(<.001)	0.40(<.001)	0.48(<.001)	−0.49(<.001)	0.16(<.001)	0.16(<.001)	0.09(<.001)	0.04(0.12)	1						
Separated	0.61	−0.59	0.47	0.54	−0.24	0.48	0.48	−0.32	−0.30	0.55	1					
	(<.001)	(<.001)	(<.001)	(<.001)	(<.001)	(<.001)	(<.001)	(<.001)	(<.001)	(<.001)						
Divorced	0.27	−0.26	0.31	0.36	−0.21	0.36	0.32	−0.18	−0.17	0.36	0.42	1				
	(<.001)	(<.001)	(<.001)	(<.001)	(<.001)	(<.001)	(<.001)	(<.001)	(<.001)	(<.001)	(<.001)					
Poverty level	0.29	−0.26	0.61	0.63	0.12	0.75	0.73	−0.61	−0.53	0.37	0.58	0.36	1			
	(<.001)	(<.001)	(<.001)	(<.001)	(<.001)	(<.001)	(<.001)	(<.001)	(<.001)	(<.001)	(<.001)	(<.001)				
Receives public assistance	0.31	−0.28	0.58	0.58	0.06	0.70	0.69	−0.57	−0.50	0.30	0.55	0.38	0.76	1		
	(<.001)	(<.001)	(<.001)	(<.001)	(0.04)	(<.001)	(<.001)	(<.001)	(<.001)	(<.001)	(<.001)	(<.001)	(<.001)			
Urban	0.57	−0.60	0.17	0.26	−0.78	−0.10	−0.17	0.40	0.34	0.6	0.36	0.32	0.03	0.06	1	
	(<.001)	(<.001)	(<.001)	(<.001)	(<.001)	(<.001)	(<.001)	(<.001)	(<.001)	(<.001)	(<.001)	(<.001)	(0.24)	(0.03)		
Rural	−0.56	0.61	−0.16	−0.25	0.78	0.11	0.18	−0.39	−0.33	−0.61	−0.35	−0.31	−0.02	−0.05	−0.99	1
	(<.001)	(<.001)	(<.001)	(<.001)	(<.001)	(<.001)	(<.001)	(<.001)	(<.001)	(<.001)	(<.001)	(<.001)	(0.40)	(0.06)	(<.001)	

^1^Unemployed, ^2^Agricultural employment, ^3^No high school diploma, ^4^Bachelor’s degree, ^5^Graduate degree, ^6^Separated, ^7^Receives public assistance.

### Socioeconomic determinants of geographic distribution of campylobacteriosis risk

The majority of the socioeconomic factors investigated for univariate (simple) associations with campylobacteriosis risk had highly significant (p<0.001) associations with campylobactriosis risk (Table 4). Due to the presence of significant overdispersion (implying that ordinary Poisson models were inappropriate for these data) and the fact that the negative binomial models fit the data better than the ordinary Poisson models (as evidenced by the lower AICs of the negative binomial models), only the results of the negative binomial models are presented.

**Table 4:** Results of assessment of univariate (simple) associations between campylobacteriosis risk and selected socioeconomic factors

**Category**	**Variable (proportion of census tract population)**	**Estimate (95% Confidence Interval)**	**SE**^**1**^	**P-value**	**AIC**^**2**^
Race & Nationality	Black or African American	−0.0133 (−0.0158,-0.0108)	0.0013	0.0001	5972
	White	0.0127 (0.0103, 0.0152)	0.0012	0.0001	5977
	Asian	0.0265 (−0.0143, 0.0672)	0.0204	0.2037	6072
	Hispanic/Latino	−0.0065 (−0.0278, 0.0148)	0.0109	0.5499	6074
	American Indian/Alaskan	0.3575 (−0.0490, 0.7641)	0.2074	0.0843	6071
Employment	Unemployed	−0.0902 (−0.1170,-0.0634)	0.0137	0.0001	6030
	Service occupation	−0.0291 (−0.0403,-0.0178)	0.0058	0.0001	6049
	Agriculture: forestry, fishing, hunting & mining	0.0585 (0.0317, 0.0854)	0.0137	0.0001	6055
	Farming Industry	0.0278 (0.0367,-0.0442)	0.0999	0.4485	6074
	Disability (age 21–64)	−0.0169 (−0.0253,-0.0086)	0.0043	0.0001	6058
	Armed forces	−0.0462 (−0.0779,-0.0144)	0.0162	0.0043	6065
Education	No High school diploma	−0.0097 (−0.0188,-0.0006)	0.0046	0.0373	6070
	Bachelor degree	0.0122 (0.0049, 0.0195)	0.0037	0.0011	6069
	Graduate/Professional Degree	0.0159 (0.0054, 0.0265)	0.0054	0.0031	6070
Marital status	Never married	−0.0134 (−0.0193, -0.007)	0.0030	0.0001	6055
	Separated	−0.1560 (−0.1909, -0.1211)	0.0178	0.0001	6000
	Divorced	−0.0390 (−0.0570, -0.020)	0.0092	0.0001	6056
	Widow	−0.0559 (−0.0776, -0.0342)	0.0111	0.0001	6049
Poverty, Public Assistance & Urbanicity	Below poverty level	−0.0198 (−0.0264,-0.0132)	0.0034	0.0001	6041
	Receives public assistance	−0.0458 (−0.0627,-0.0289)	0.0086	0.0001	6047
	Urban	−0.2573 (−0.4133,-0.1013)	0.0796	0.0012	6064
	Rural	0.2573 (0.1013, 0.4134)	0.0796	0.0012	6064

^1^ Standard Error.

^2^Akaike’s Information Criterion.

**Figure 3:**
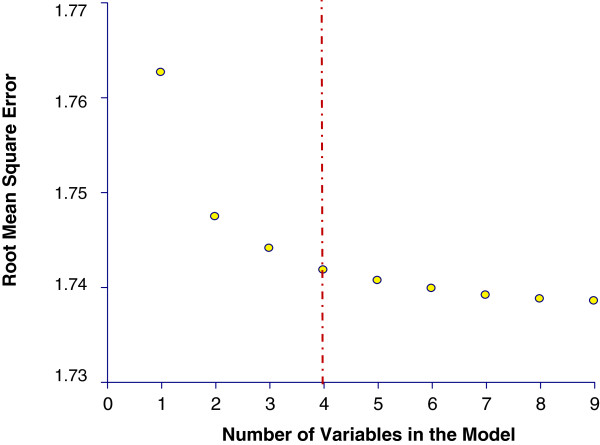
**McHenry’s All Possible variable selection procedure scree plot demonstrating root mean square error improvement in the top model combinations.** Improvement is optimized with four variables (dashed red line).

McHenry’s All Possible variable selection, used to identify the most optimal parsimonious models, revealed an exponential drop and stabilization of the root mean square error with four variables in the model (Figure 3). This implies that the most optimal and parsimonious models were those that had four explanatory variables. Thus, the final competing models included four explanatory variables. Models 1 & 2 had the best fit as evidenced by the fact that they had the lowest AICs. Therefore, these models were used to compare the 4 modeling approaches (negative binomial, spatial lag, global GWR and local GWR) investigated in this study. Both negative binomial models 1 and 2 showed that the risk of campylobacteriosis tended to be lower in census tracts that had higher proportions of blacks and unemployed populations (Table 5). However, campylobacteriosis risks were significantly higher in census tracts that had high proportions of the population with no high school diploma (Table 5). Additionally, in Model 1, census tracts with high proportions of the population living in urban areas tended to have higher risk of campylobacteriosis, whereas in Model 2, the risk of campylobacteriosis was lower in census tracts with high divorce rate.

**Table 5:** Comparison of negative binomial, spatial lag, global and local geographically weighted Poisson models

	**Model type with Coefficient estimates (p-values)**				
	**Negative Binomial Model**	**Spatial Lag Model**	**Global Poisson GWR**^**1**^**Model**	**Local Poisson GWR**^**1**^**Model**	
**Model 1:**				**Min**	**Max**
Intercept	−7.164 (0.0001)	1.716 (0.000)	- 7.155 (0.000)	−8.187	-6.247
Black Race	−0.015 (0.0001)	−0.169 (0.001)	−0.014 (0.001)	−0.0487	0.0218
No diploma	0.021 (0.0004)	−0.012 (0.112)	0.003 (0.003)	−0.0553	0.0533
Unemployed	−0.041 (0.0141)	−0.030 (0.112)	−0.014 (0.009)	−0.1866	0.0851
Urban	0.235 (0.0154)	0.357 (0.014)	0.186 (0.055)	−0.4526	0.9321
**Model 2:**					
Intercept	- 6.73 (0.0001)	1.80 (0.000)	- 6.85 (0.000)	−7.71	-4.905
Black Race	−0.0129 (0.0001)	−0.093 (0.000)	−0.012 (0.001)	−0.0161	0.0311
No diploma	0.0175 (0.0009)	−0.018 (0.011)	0.000 (0.003)	−0.0650	0.0882
Unemployed	−0.0330 (0.0433)	−0.026 (0.179)	−0.010 (0.008)	−0.1847	0.0752
Divorced	−0.0260 (0.006)	−0.004 (0.733)	−0.016 (0.005)	−0.2485	0.0382

^1^ Geographically Weighted Regression.

Assessment of Pearson’s standardized residuals from the negative binomial models showed evidence of residual positive spatial autocorrelation (Moran’s I:0.083, p=0.001). This implies that, although the negative binomial model was better than the ordinary Poisson model, it still has residual spatial autocorrelation and thus did not totally eliminate spatial autocorrelation. The presence of significant spatial autocorrelation in the residuals requires use of an appropriate spatial model.

### Comparison of the modeling approaches

Based the AIC goodness-of-fit statistic for comparing models, the model with the lowest AIC statistic is the one with the best model fit. Using this criterion to compare the 4 modeling approaches used to fit model 1, the local Poisson GWR model had the best fit (AIC=3344) followed by the global Poisson GWR (AIC=4854), spatial lag (AIC=4908) and lastly negative binomial model (AIC=5962). A similar pattern was observed for model 2 with local Poisson GWR again having the best fit (AIC=3244), followed again by global GWR (AIC=4860), then spatial lag (AIC=4914) and finally negative binomial (AIC=5960). The overall best fitting model is local GWR model 2 which had the lowest AIC of 3244.

It is important to stress that both local Poisson GWR models 1 and 2 showed evidence of non-stationarity of all the regression coefficients. This is evidenced by the fact that the interquartile ranges of the local regression coefficients were all larger than twice the standard errors of the regression coefficients of the global Poisson GWR model (Table 6). This implies that the regression coefficients of each of the variables included in the local GWR models were not constant but changed across the census tracts in the study area. The implication of this is that, the strength of the associations between campylobacteriosis risk and each of the explanatory variables vary depending on the spatial location. Thus, assuming a causal relationship, the effects of the determinants are not constant across the study area but are heavily dependent on the geographical location [32].

**Table 6:** Assessment of the stationarity of the local Geographically Weighted Regression (GWR) Model coefficients

**Model 1:**	**Global Poisson GWR**^**1**^**SE**^**2**^	**Global Poisson GWR**^**1**^**2xSE**^**2**^	**Local Poisson GWR**^**1**^**IQR**^**3**^	**Is Regression Coefficient Non-Stationary?**
Black Race	0.001	0.002	0.015	Yes
No diploma	0.003	0.006	0.028	Yes
Unemployed	0.009	0.018	0.068	Yes
Urban	0.046	0.092	0.469	Yes
**Model 2:**				
Black Race	0.001	0.002	0.016	Yes
No diploma	0.003	0.006	0.015	Yes
Unemployed	0.009	0.018	0.067	Yes
Divorced	0.005	0.010	0.033	Yes

^1^ Geographically Weighted Regression.

^2^ Standard error of the global GWR model.

^3^ Interquartile local coefficient estimate range.

The spatial autoregressive coefficients (ρ) of both the spatial lag model 1 (ρ=0.622; p<0.001) and spatial lag model 2 (ρ=0.655; p<0.001) were significantly greater than 0 confirming the fact that there was significant spatial clustering in the data. Statistical significance of the these autoregressive coefficients also imply that the non-spatial models (i.e. the negative binomial models are inappropriate for these data). This conclusion is further supported by the results of the assessment of the goodness-of-fit of the models which revealed that the negative binomial models had the worst fit.

**Figure 4:**
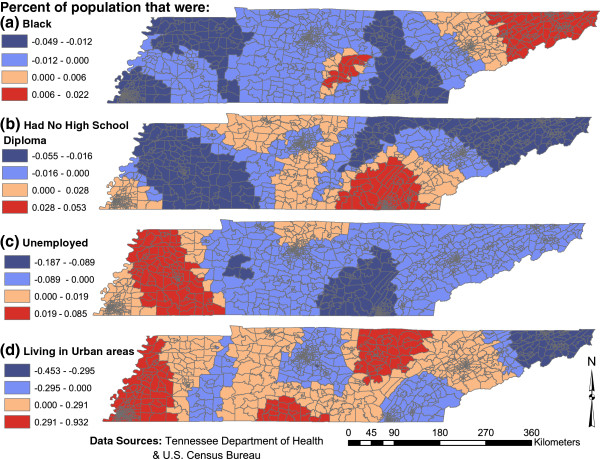
Model 1 geographically weighted parameter estimates of the significant socioeconomic determinants of campylobacteriosis risk in Tennessee.

**Figure 5:**
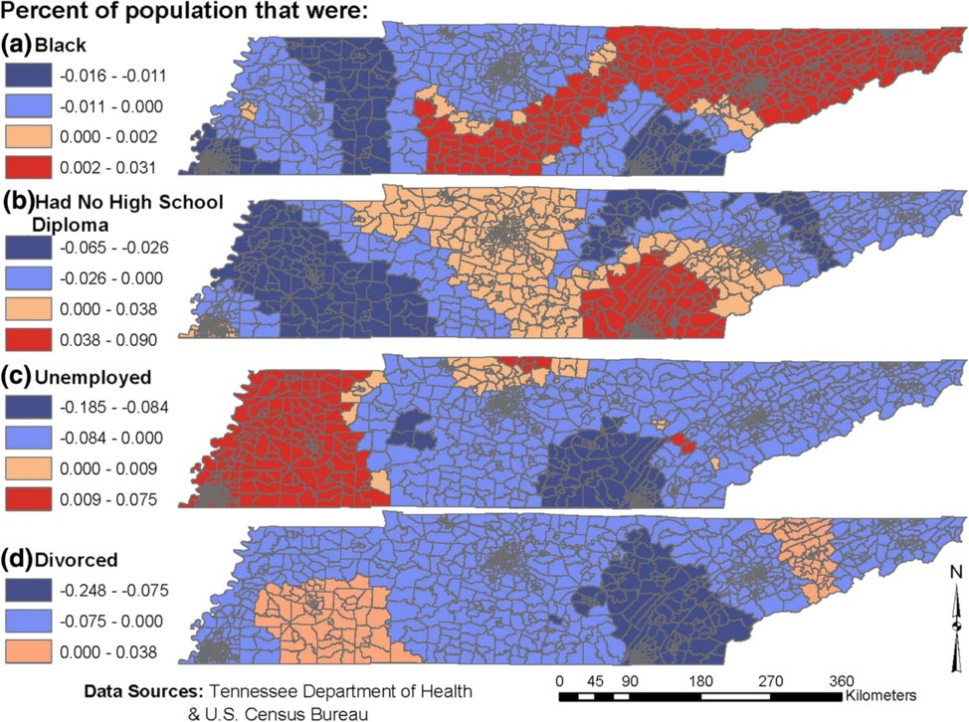
Model 2 geographically weighted parameter estimates of the significant socioeconomic determinants of campylobacteriosis risk in Tennessee.

The spatial patterns of the local GWR regression coefficients of the explanatory variables common to models 1 and 2 are shown in Figures 4 and 5, respectively, and the statistical evidence of their non-stationarity is shown on Table 6. On both local Poisson GWR models 1 and 2, campylobacteriosis risk tended to be higher in census tracts that had high proportions of individuals with no high school diploma and these census tracts were mainly located in southeast Tennessee. Areas where lack of high school diploma had the strongest positive association with campylobacteriosis risk tended to have relatively high proportions of black population (18.4%). By contrast, areas where lack of high school education had the strongest negative association with campylobacterisis risk had relatively few blacks (only 8.4%). Moreover, areas where lack of high school diploma had the strongest positive association with campylobacteriosis risk tended to have a low (29%) proportion of the population living in rural areas (i.e. they were mainly urban areas). On the other hand, areas where lack of high school diploma had a negative association with campylobacteriosis risk tended to be more rural with as high as 61% of the population living in rural areas. Thus, assuming a causal relationship, it appears that low education has a more marked impact on increasing risk of campylobacteriosis in the urban than in the rural areas. It is also worth noting that that areas where lack of high school diploma had the strongest positive association (strong risk factor) tended to have relatively high mean income ($46,000) compared to those where it had the strongest negative association with campylobacteriosis risk where the mean income was $40,300.

The strongest positive association between high unemployment rate and campylobacteriosis risk was observed in the western third of the state (Figures 4c &5c). These areas tended to be in the rural having, on average, 62% of the population living in rural areas. On the other hand, areas where high unemployment rate had a negative association with campylobacteriosis risk tended to be urban with only 36.9% of the population living in rural areas.

In some urban centers, such as Memphis and Knoxville, and their surrounding areas, campylobacteriosis risk was high, whereas in other urban centers, such as Nashville, the risk was low. With regard to the geographic disparities in distribution of the association between campylobacteriosis risk and urbanicity, the areas with the strongest positive association (Figure 4d) between urbanicity and campylobacteriosis risk tended to have a relatively higher percentage of black (38.9%) compared to areas which had the strongest negative association between urbanicity and campylobacteriosis risk (Figure 4d) that had only 2.5% of black population.

Areas that had the strongest positive association between campylobacteriosis risk and divorce rate (Figure 5d) tended to be in the rural where, on average, 75.1% of the population lived in rural areas. By contrast, areas that had the strongest negative association between campylobacterisis risk and divorce rate tended to be urban where only an average of 34.4% of the population was rural.

## Discussion

Although past studies have investigated associations between socioeconomic factors and campylobacteriosis risk and others have reported that campylobacteriosis risk varies geographically [7,33], to our knowledge, no studies have used local GWR approaches to investigate the geographic variations of the association between campylobacteriosis risk and socioeconomic factors. Thus, the current study is, in part, intended to fill this knowledge gap. The modeling approaches used in this study (i.e. local GWR) are novel and provide powerful tools to epidemiological investigations and should therefore be applied to many diseases throughout the world. Although local GWR models offer insight into socioeconomic risk factors and their complex relationships with health outcomes not many studies have used them. We believe that these spatial variations in regression coefficients need to be investigated to ensure that appropriate disease control programs are used regardless of the disease of interest and the geographical areas concerned.

The global multivariable models in our study showed that census tracts with high proportions of the population that were black, unemployed and divorced tended to consistently have a lower risk of campylobacteriosis, whereas those with high proportions of the population living in urban areas, and with no high school diploma had a higher risk of campylobacteriosis. In contrast, local Poisson GWR models revealed a diverse range of regression coefficients for the associations between campylobacteriosis risk and the socioeconomic determinants across the study area. Thus, since the regression coefficients of the determinants ranged from negative to positive over the study area, global models are inaccurate and unreliable. This complex spatial heterogeneity in the associations between socioeconomic factors and campylobacteriosis risk explains: (i) why local Poisson GWR models outperformed negative binomial, spatial lag and global Poisson GWR models and (ii) how global models mask the true nature of the relationships between determinants and campylobacteriosis risk. These findings imply that the strength of association between a determinant and disease changes by location and this needs to be factored in disease control programs since a factor may be a more important determinant of disease in some areas and not others.

Local Poisson GWR results identified spatial patterns for some of the spatially varying coefficients in this study. For instance, positive associations were observed between high campylobacteriosis risk and urbanicity in areas that tended to have low education attainment and high proportion of blacks. Similarly, areas which had positive association between high campylobacteriosis risk and high divorce and unemployment rates tended to be rural. These patterns support the hypothesis that the reasons for the differences in campylobacteriosis risk vary geographically across the study area. In fact, several studies have reported that variation in the risk of campylobacteriosis may be due to regional differences in the distribution of socioeconomic risk factors, as well as unknown or underlying regional characteristics [4,14,33,34]. For example, wealthy and highly educated populations might acquire campylobacteriosis through exposure to undercooked foods in restaurants or contaminated outdoor environments while vacationing, whereas populations living in poverty or with lower levels of education attainment may be exposed through poor food handling at home. Regional variation in how underlying socioeconomic characteristics influence the parameter estimates of high risk areas warrant further investigation at a local level. Suffice it to say that the global models do not provide the true nature of the relationships which sometimes varies from negative association in some areas to positive associations in others. This has practical implications in disease control because a one-size-fits-all strategy (which would be used if results of global models are used) is not appropriate since local Poisson GWR reveals that certain determinants may be more important in some areas than others. Thus, health planning and service provision need to use a needs-based approach based on empirical data such as these.

The fact that areas where lack of high school diploma had the strongest positive association with disease risk tended to have relatively high mean income could be due to the income disparities between the urban and rural areas where the urban populations tend to earn slightly more than the very rural areas. On the other hand, the fact that areas where lack of high school diploma had a negative association with campylobacteriosis risk tended to be rural seems to suggest that low level of education may have a higher impact on risk of campylobacteriosis in urban than rural areas probably due to higher cost of living in the more urban centers that would potentially force low education and low income population to have much poorer living conditions in the cities than in the rural areas. Poorer living conditions would then inevitably increase the risk of campylobacter infections in these populations. The observed lower risk of campylobacteriosis in census tracts with high proportions of blacks and unemployed population might be a reflection of under reporting rather than a lower disease burden in these communities.

The association between campylobacteriosis risk and the proportion of the census tract population living in urban areas showed the largest spatial variation, as evidenced by the coefficient range (−0.453 to 0.932) across the study area. Campylobacteriosis risk factors have been shown to differ between rural and urban environments due to different direct and indirect exposure opportunities [11]. Typical rural exposures include poultry and farm animals, unpasteurized milk and contaminated surface waters [35-38]. Yet exposure to *Campylobacter* infection by poultry and farm animals is not limited to rural areas, as animal products are processed and distributed at varying distances from their source, and contamination may result from processing plants located in urban areas. This source of environmental contamination from the food industry is an underlying risk factor which should be investigated locally in rural and urban regions.

In our study, local Poisson GWR models had better statistical fit (lower AIC’s) than the global models investigated. These findings are similar to those of several other studies that have compared the performance of local GWR and global regression methods in investigating associations between disease and risk factors [15,16,21,39]. For example, after modeling determinants of drug resistant tuberculosis, Liu et al. reported that the local GWR model had a much better fit (AIC=395) than the global regression model (AIC=471). Moreover, the GWR model had an increase of over 15% in explaining the variation in the outcome. Gilbert and Chakraborty and Cheng et al. reported spatial variability in regression coefficients and found improvements of over 10% in R^2^, and decreases in AIC (over 70 units), respectively, for local compared to global models [16,39]. Although local GWR modeling is relatively new, their results convincingly indicate that local spatial characteristics can have a profound effect on regression coefficients and statistical significance of variables [40]. Geographic disparities in risk should therefore be investigated at local levels to: (i) capture regional differences in the nature of the relationship between risk factors and disease outcome, (ii) avoid misleading inferences and conclusions from global models, and (iii) better inform disease control programs.

One of the primary goals of the U.S. Department of Health and Human Services is to eliminate health disparities associated with socioeconomic status and geographic location [41]. Accurate measurement and reporting of health disparities has important implications for decision-making and policy implementation at a local, national and international levels [42]. Quantifying the effects of socioeconomic factors should be prioritized and approached in an interdisciplinary and collaborative manner using methodologically sound techniques [8,17]. Therefore, advanced analytical techniques such as GWR, which incorporate geography into epidemiological studies in novel ways, need to be more widely accessible to researchers and epidemiologists globally.

The complexity of the relationship between geography and socioeconomic status creates a difficult task for public health professionals. Health disparities are likely to change empirically as societal conditions change over time and space [9]. Populations move and become diverse, altering social and ethnic demographics and disease patterns [8]. Local GWR modeling strategies address these issues more efficiently by helping to identify differences in the strengths of association between determinants and health outcomes across areas [17]. By adopting strategies that target known high risk socioeconomic groups, limited and precious resources can be more efficiently allocated and policy and planning can better target regional public health needs.

Although approximately 20% of the disease data in this study were eliminated due to missing information or inaccuracy of residential addresses, the spatial distribution of cases with missing data was similar to those whose data were complete. This suggests that the missing data were randomly distributed, and therefore not likely to have biased the results of our study. The impact of using the year 2000 US Census population data as denominators (for computing campylobacteriosis risk) and as offsets (for the ordinary Poisson and negative binomial models) for a study spanning 17 years is not known. However, it was the only population data available for the study period and therefore offered the best representation of both population and socioeconomic data.

Suffice it to say that the local GWR methods used in this study are quite novel and would significantly add to the spatial epidemiologist’s toolbox when investigating determinants of geographical disparities of health outcomes. Thus, although the specific results of this study may not be generalizable to other regions in the world, the methods used and results obtained are eye openers to spatial epidemiologists across the globe that deeper insights are obtained when local GWR models are used to investigate determinants of health since the magnitude of the impact of determinants vary by geographical location. This is important information that can be used by health planners and service providers to ensure that resources are better allocated to improve health outcomes. There is no doubt that these tools need to be incorporated in routine investigations by epidemiologists and decision makers interested in addressing issues related to health disparities so as to improve health outcomes for all.

## Conclusion

The international significance of the findings from this work is that they highlight the fact that global regression strategies, frequently used to investigate determinants of geographic disparities in disease distribution, generally tend to mask the true nature of the relationship between the outcome and explanatory variables. Since local GWR models estimate a regression coefficient for each location in a study area, they are able to more powerfully reveal the geographic differences in the associations between the explanatory variables and the outcome/disease. Thus, the information obtained provides critical empirical evidence to health planners and public health professionals to guide health planning and disease control programs. Since the regression coefficients change based on geographical location, it implies that a determinant of disease may be a more important risk factor in one location and not other locations. Incorporating this information in health planning and service provision ensures that health professionals do not use a “one-size-fits-all” approach but instead the planning and provision of services would be guided by the needs of the areas as evidenced by the local regression coefficients of specific disease determinants. Thus, local GWR regression models should be an important addition to the toolbox of public health epidemiologists globally. This tool would allow them to assess how the impact of different determinants of disease outcomes vary by geographical location which information would greatly improve the decision making process in relation to allocation of resources for disease control programs.

## Abbreviations

GWR: Geographically weighted regression; FoodNet: Foodborne Diseases Active Surveillance Network; SES: Socioeconomic status; AIC: Akaike’s Information Criterion.

## Competing interests

The authors declare that they have no competing interests.

## Authors’ contributions

JW conceived the original idea, participated in study design, performed data analysis and drafted the manuscript. AO was involved in conception of research idea, study design, data analysis and extensive editing of the manuscript. BR assisted with study design and manuscript preparation. JD provided data for the study and assisted with manuscript preparation. All authors read and approved the final manuscript.
